# Cigarette smoking complements the prognostic value of baseline plasma Epstein-Barr virus deoxyribonucleic acid in patients with nasopharyngeal carcinoma undergoing intensity-modulated radiation therapy: a large-scale retrospective cohort study

**DOI:** 10.18632/oncotarget.7609

**Published:** 2016-02-23

**Authors:** Jia-Wei Lv, Yu-Pei Chen, Guan-Qun Zhou, Ling-Long Tang, Yan-Ping Mao, Wen-Fei Li, Rui Guo, Ai-Hua Lin, Jun Ma, Ying Sun

**Affiliations:** ^1^ Department of Radiation Oncology, Sun Yat-sen University Cancer Center, State Key Laboratory of Oncology in South China, Collaborative Innovation Center for Cancer Medicine, Guangzhou, People's Republic of China; ^2^ Department of Medical Statistics and Epidemiology, School of Public Health, Sun Yat-sen University, Guangzhou, People's Republic of China

**Keywords:** baseline plasma EBV DNA, cigarette smoking, intensity-modulated radiation therapy, nasopharyngeal carcinoma, prognostication

## Abstract

We evaluated the combined prognostic value of cigarette smoking and baseline plasma Epstein-Barr virus deoxyribonucleic acid (EBV DNA) in patients with nasopharyngeal carcinoma (NPC) treated with intensity-modulated radiation therapy (IMRT). Of consecutive patients, 1501 with complete data were eligible for retrospective analysis. Smoking index (SI; cigarette packs per day times smoking duration [years]), was used to evaluate the cumulative effect of smoking. Primary endpoint was overall survival (OS); progression-free survival (PFS), distant metastasisfree survival (DMFS) and locoregional relapse-free survival (LRFS) were secondary end-points. Both cigarette smoking and baseline plasma EBV DNA load were associated with poorer survival (*P*<0.001). Patients were divided into four groups: low EBV DNA and light smoker (LL), low EBV DNA and heavy smoker (LH), high EBV DNA and light smoker (HL), and high EBV DNA and heavy smoker (HH). The respective 5-year survival rates were: OS (93.1%, 87.2%, 82.9%, and 76.3%, *P*<0.001), PFS (87.0%, 84.0%, 73.9%, and 64.6%, *P*<0.001), DMFS (94.1%, 92.1%, 82.4%, and72.5%, *P*<0.001), and LRFS (92.8%, 92.4%, 88.7%, and 84.0%, *P*=0.012).OS and PFS were significantly different between the LH and HL groups and HL and HH groups, but not LL and LH groups (pairwise comparisons). The combined risk stratification remained an independent prognostic factor for all endpoints (all *P*_trend_<0.001; multivariate analysis). Both cigarette smoking and baseline plasma EBV DNA were independent prognostic factors for survival outcomes. Combined interpretation of EBV DNA with smoking led to the refinement of the risks stratification for patient subsets, especially with improved risk discrimination in patients with high baseline plasma EBV DNA.

## INTRODUCTION

Nasopharyngeal carcinoma (NPC) is a squamous cell carcinoma that originates from the epithelial lining of the nasopharynx and is endemic in southern China and Southeast Asia, where the prevalence is approximately 50 cases per 100,000 individuals. [1, 2] Radiotherapy (RT) is the primary treatment modality for NPC. [3] The introduction of intensity-modulated radiation therapy (IMRT) in recent years has greatly enhanced the locoregional control rate and leads to superior outcomes in NPC. The tumor-node-metastasis (TNM) staging system remains the most recognizable and commonly-used prognostic indicator for patients with NPC. [4] However, due to the heterogeneity of patient and tumor factors, even patients with identical classifications and clinical stages exhibit a variety of differing outcomes after treatment. [5] Thus, potential exists for the identification of novel prognostic factors that are directly or indirectly related to treatment outcome.

Numerous efforts have been made to discover novel potential prognostic factors in recent years. Among these, baseline plasma Epstein-Barr virus DNA (EBV DNA) has attracted increasing attention and become a major focus of research. At present, baseline plasma EBV DNA is widely assessed in clinical use for its proven ability to assist with diagnosis, risk stratification, prognostication and relapse supervision in patients with NPC. [6-8] However, even patients with the same baseline plasma EBV DNA load exhibit different treatment outcomes, which reflects the heterogeneity of NPC. [9] Hence, additional efforts should be made to explore other markers that may correlate with and complement baseline plasma EBV DNA in order to improve risk stratification and prognostication in NPC.

Among the various prognostic factors that have already been reported, cigarette smoking attracts attention due to its demonstrated relationships with NPC etiology, prognostication and EBV seropositivity. [10-12] Xu et al. [13] reported that cigarette smoke extract could induce EBV replication and promoted the EBV latent-to-lytic switch *in vitro*. Additionally, according to a 20-year follow-up study in Taiwan, heavy smokers maintained a higher EBV seropositivity rate than never smokers and light smokers and were proned to higher risk of nasopharyngeal carcinoma. [10]

Given the evidence above, we conducted a large-scale retrospective study to reevaluate the prognostic significance of cigarette smoking and baseline plasma EBV DNA in patients with NPC undergoing IMRT, and investigated the combined value of these factors for risk stratification and prognostication with the aim of improving the individualized treatment of patients with NPC receiving IMRT.

## RESULTS

### Patient characteristics, patterns of failure and survival

The characteristics of all 1501 patients are summarized in Table 1. The median follow-up for the entire group was 48.4 months (range: 1.3-76.4 months). The clinical stage distribution for the whole cohort was: stage I, 73(4.9%), stage II, 312(20.8%), stage III, 709(47.2%); and stage, IV 407(27.1%). In total, 195(13.0%) patients received RT alone, while 1306(87.0%) patients received concurrent chemoradiotherapy ± neoadjuvant chemotherapy/adjuvant chemotherapy (CCRT ± NACT/AC). Of the 1116 patients with locally-advanced NPC, 1057/1116 (94.7%) received chemotherapy. All patients in this cohort received IMRT.

**Table 1:** Clinical characteristics of the 1501 patients with nasopharyngeal carcinoma

	Total	Smoking index		
		≤ 6.5 pack/years	> 6.5 pack/years	
Characteristic	(n=1501)	(n=1030)	(n=471)	*P*-value
	No. (%)	No. (%)	No. (%)	
Age				<0.001
≤45	785(52.3)	596(75.9)	189(24.1)	
>45	716(47.7)	434(60.6)	282(39.4)	
Gender				<0.001
Male	1114(74.2)	649(58.3)	465(41.7)	
Female	387(25.8)	381(98.4)	6(1.6)	
Histological type				0.49
I	8(0.5)	7(87.5)	1(12.5)	
II	77(5.1)	54(70.1)	23(29.9)	
III	1416(94.3)	969(68.6)	447(31.4)	
T classification^†^				0.01
T1	261(17.4)	195(74.7)	66(25.3)	
T2	234(15.6)	162(69.2)	72(30.8)	
T3	720(48.0)	497(69.0)	223(31.0)	
T4	286(19.1)	176(61.5)	110(38.5)	
N classification^†^				0.103
N0	233(15.5)	173(74.2)	60(25.8)	
N1	881(58.7)	606(68.8)	275(31.2)	
N2	239(15.9)	157(65.7)	82(34.3)	
N3	148(9.9)	94(63.5)	54(36.5)	
Stage^†^				0.002
I	73(4.9)	61(83.6)	12(16.4)	
II	312(20.8)	220(70.5)	92(29.5)	
III	709(47.2)	494(69.7)	215(30.3)	
IV	407(27.1)	255(62.7)	152(37.3)	
Chemotherapy				0.092
Yes	1306(87.0)	886(67.8)	420(32.2)	
No	195(13.0)	144(73.8)	51(26.2)	
EBV DNA*				0.022
Low	849(56.6)	603(71.0)	246(29.0)	
High	652(34.4)	427(65.5)	225(34.5)	

Abbreviations: EBV DNA = Epstein-Barr virus deoxyribonucleic acid.

† According to the 7th edition of the American Joint Committee on Cancer. Low EBV DNA is a baseline plasma EBV DNA level ≤ 4000 copies/mL; high EBV DNA, > 4000 copies/mL. Light smoker is a smoking index ≤ 6.5 pack-years; heavy smoker, > 6.5 pack-years.

The proportions of light smokers and heavy smokers were 471/1501 (31.4%) versus 1030/1501 (68.6%). There was no significant difference in the distribution of histological types, chemotherapy strategies or N classification when the patients were stratified by cigarette smoking. However, significant differences were observed in terms of age, gender, T category, clinical stage and baseline plasma EBV DNA.

Of the entire cohort, 126 (8.4%) patients developed local recurrences. 169(11.3%) patients developed distant metastases. 165 (11.0%) patients died. For the entire cohort, the 5-year OS, PFS, DMFS, and LRFS rates were 89.0%, 81.8%, 88.7%, and 91.6% respectively.

### Correlation between baseline plasma EBV DNA and cigarette smoking, and their prognostic value in NPC patients undergoing IMRT

**Figure 1:**
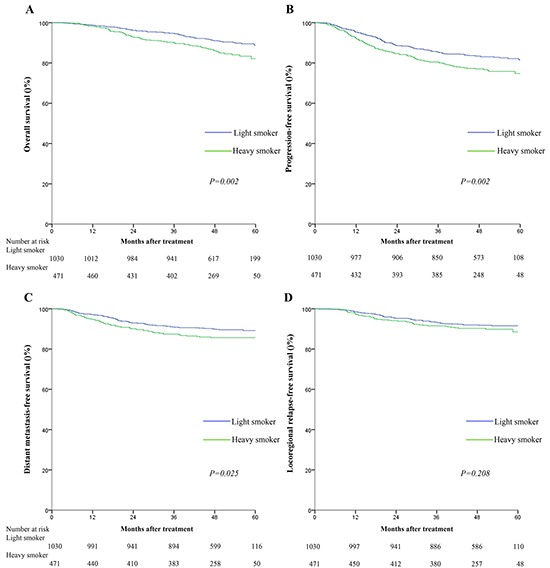
Kaplan-Meier survival curves for overall survival A. progression-free survival B. distant metastasis-free survival C. and locoregional relapse-free survival D. for the 1501 patients with NPC stratified as heavy smokers and light smokers All categories are based on the 7^th^ edition of the Union for International Cancer Control/American Joint Committee on Cancer staging system. Smoking index was calculated by multiplying cigarette packs per day and smoking time (years). The cutoff value for smoking index was≤6.5 pack-years.

Light smokers and heavy smokers had significantly different 5-year OS (88.8% vs. 82.1%, *p=*0.002), PFS (81.5% vs. 74.7%, *p*=0.002) and DMFS rates (89.2% vs. 85.7%, *p*=0.025); however, LRFS was not significantly different between light smokers and heavy smokers (91.5% vs. 88.5%, *p*=0.208; Figure 1). Multivariate analyses indicated that the cumulative effect of smoking remained an independent predictor for OS (HR=1.38, 95%CI:1.01-1.88) and PFS (HR=1.28, 95%CI:1.02-1.60), but not DMFS or LRFS, after adjustment (Table 2).

**Table 2:** Cox proportional hazards analyses of cigarette smoking in the 1501 patients with NPC undergoing IMRT

Endpoint	Variable	HR	HR(95%CI)	*P*-value*
OS	N classification (N2-3 vs. N0-1)	2.50	1.83-3.40	<0.001
	T classification (T3-4 vs. T1-2)	2.00	1.32-3.00	0.001
	Age (>45 vs. ≤45 years)	1.75	1.27-2.41	0.001
	Smoking (heavy vs. light)	1.38	1.01-1.88	0.047
PFS	N classification (N2-3 vs. N0-1)	2.01	1.58-2.56	<0.001
	Chemotherapy(Yes vs. No)	1.66	1.02-2.70	0.041
	Age (>45 vs. ≤45 years)	1.31	1.03-1.67	0.024
	Smoking (heavy vs. light)	1.28	1.02-1.60	0.031
DMFS	N classification (N2-3 vs. N0-1)	2.85	2.10-3.87	<0.001
	Smoking (heavy vs. light)	NS	---	---
LRFS	N category (N2-3 vs. N0-1)	NS	---	---
	Smoking (heavy vs. light)	NS	---	---

Abbreviations: CI = confidence interval; DMFS = distant metastasis-free survival; EBV DNA = Epstein-Barr virus deoxyribonucleic acid; HR = hazard ratio; IMRT = intensity-modulated radiotherapy; LRFS = locoregional relapse-free survival; NPC = nasopharyngeal carcinoma; NS = not significant; OS = overall survival.

Light smoker is a smoking index ≤ 6.5 pack-years; heavy smoker, > 6.5 pack-years.

* The following parameters were included in the Cox proportional hazards model multivariate analysis with backward elimination: age (> 45 vs. ≤ 45 years), gender (female vs. male), smoking (heavy vs. light), T category (T3–4 vs. T1-2), N category (N2–3 vs. N0-1) and chemotherapy (yes vs. no).

**Figure 2:**
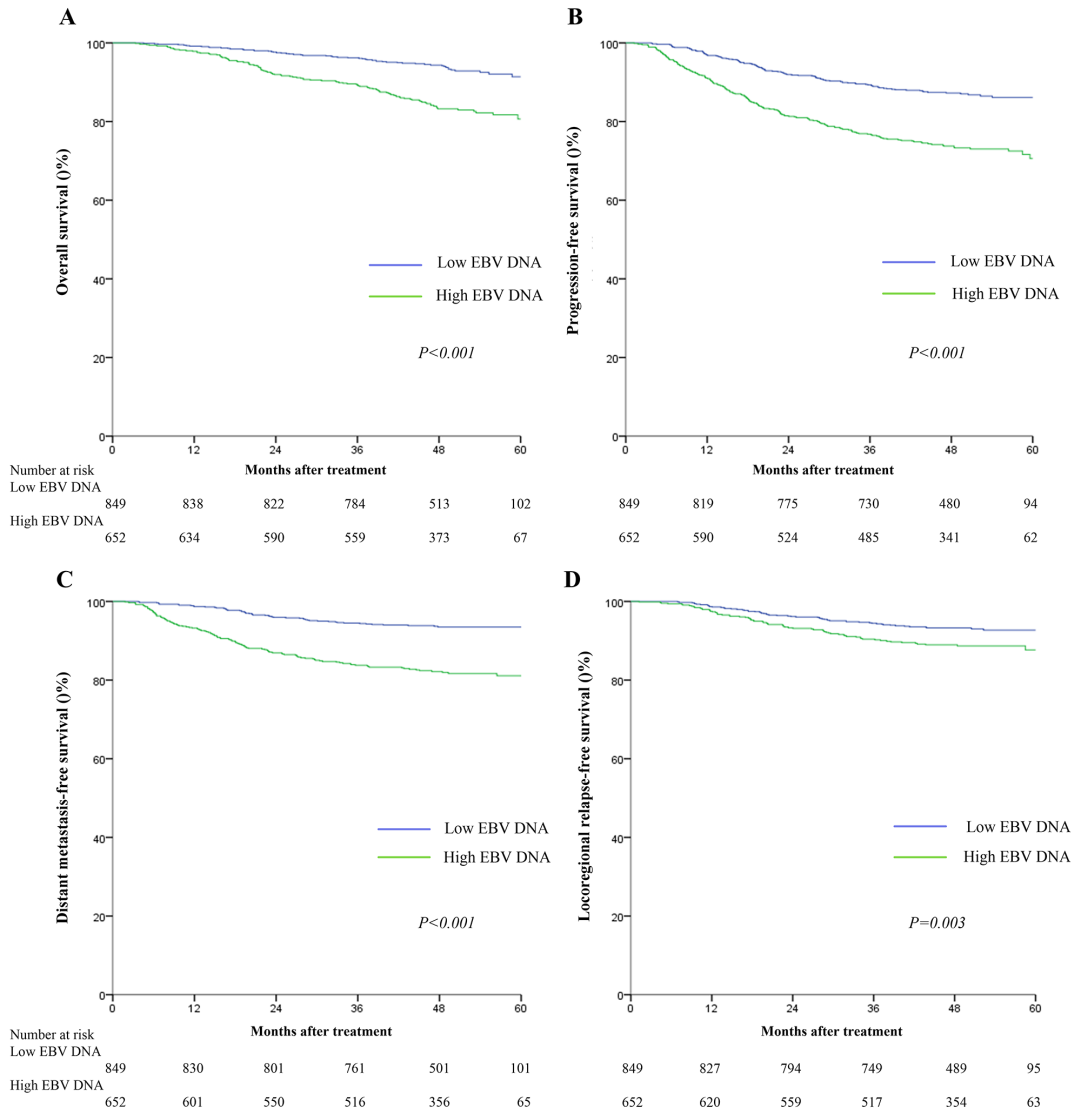
Kaplan-Meier survival curves for overall survival A. progression-free survival B. distant metastasis-free survival C. and locoregional relapse-free survival D. for the 1501 patients with NPC stratified as the high and low baseline plasma EBV groups All categories are based on the 7^th^ edition of the Union for International Cancer Control/American Joint Committee on Cancer staging system. The cutoff value for the baseline plasma EBV DNA load was≤4000 copies/mL.

Compared to patients with high baseline plasma EBV DNA (> 4000 copies), patients with low baseline plasma EBV DNA (≤4000 copies) had significantly higher 5-year OS (91.4% vs. 80.6, *P* < 0.001), PFS (86.1% vs. 70.6%, *P* < 0.001), DMFS (93.5% vs. 81.1%, *P* < 0.001), and LRFS rates (92.7% vs. 87.7%, *P*=0.003; Figure 2). In multivariate analyses, baseline plasma EBV DNA remained an independent, negative prognostic factor for OS (HR=1.97, 95%CI: 1.42-2.75), PFS (HR=1.91, 95%CI: 1.49-2.45), DMFS (HR=2.55, 95%CI: 1.82-3.55) and LRFS (HR=1.68, 95%CI: 1.18-2.39) after adjustment (Table 3).

**Table 3:** Cox proportional hazards analyses for baseline plasma EBV DNA in the 1501 patients with NPC undergoing IMRT

Endpoint	Variable	HR	HR(95%CI)	*P*-value*
OS	Age (>45 vs. ≤45 years)	1.80	1.32-2.47	<0.001
	T classification (T3-4 vs. T1-2)	1.97	1.31-2.95	0.001
	N classification (N2-3 vs. N0-1)	2.27	1.66-3.11	<0.001
	EBV DNA (high vs. low)	1.97	1.42-2.75	<0.001
PFS	Age (>45 vs. ≤45 years)	1.37	1.08-1.73	0.009
	N classification (N2-3 vs. N0-1)	1.79	1.41-2.29	<0.001
	EBV DNA (high vs. low)	1.91	1.49-2.45	<0.001
DMFS	N classification (N2-3 vs. N0-1)	2.51	1.84-3.42	<0.001
	EBV DNA (high vs. low)	2.55	1.82-3.55	<0.001
LRFS	EBV DNA (high vs. low)	1.68	1.18-2.39	0.004

Abbreviations: CI = confidence interval; DMFS = distant metastasis-free survival; EBV DNA = Epstein-Barr virus deoxyribonucleic acid; HR = hazard ratio; IMRT = intensity-modulated radiotherapy; LRFS = locoregional relapse-free survival; NPC = nasopharyngeal carcinoma; NS = not significant; OS = overall survival.

Low EBV DNA is a baseline plasma EBV DNA level ≤ 4000 copies/mL; high EBV DNA, > 4000 copies/mL.

* The following parameters were included in the multivariate analysis using the Cox proportional hazards model by backward elimination: age (> 45 vs. ≤ 45 years), gender (female vs. male), EBV DNA (high vs. low), T category (T3–4 vs. T1-2), N category (N2–3 vs. N0-1) and chemotherapy (yes vs. no).

As both the cumulative effect of smoking and baseline plasma EBV DNA were independent prognostic factors in patients with NPC undergoing IMRT, the Chi-square test was conducted to further investigate the relationship between cigarette smoking and baseline plasma EBV DNA. The cumulative effect of smoking correlated significantly with baseline plasma EBV DNA (*P* = 0.022); heavy smokers tended to have higher baseline plasma EBV DNA loads, whereas light smokers tended to have lower baseline plasma EBV DNA loads.

### Construction and validation of a novel risk stratification system that combines baseline plasma EBV DNA and cumulative effect of smoking

**Figure 3:**
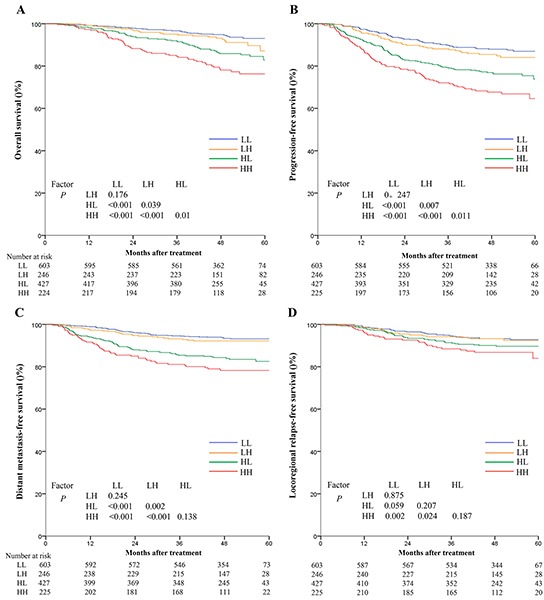
Kaplan-Meier survival curves for overall survival A. progression-free survival B. distant metastasis-free survival C. and locoregional relapse-free survival D. for the 1501 patients with NPC stratified using the combination of baseline plasma Epstein-Barr virus DNA (EBV DNA) and cumulative effect of smoking All categories are based on the 7^th^ edition of the Union for International Cancer Control/American Joint Committee on Cancer staging system. LL: low EBV and light smokers with EBV DNA≤4000 copies/mL and SI≤6.5 pack-years; LH: low EBV and heavy smokers with EBV DNA≤4000 copies/mL and SI > 6.5 pack-years; HL: high EBV and light smokers with EBV DNA > 4000 copies/mL and SI≤6.5 pack-years; HH: high EBV and heavy smokers with EBV DNA > 4000 copies/mL and SI > 6.5 pack-years.

Since cumulative effect of smoking correlated positively with baseline plasma EBV DNA, and both of these features were independent prognostic factors for survival outcomes, we constructed a novel risk stratification that combined these two markers with the aim improving prognostication in NPC. Therefore, the whole cohort was divided into four groups, as follows: low EBV DNA and light smoker (LL), low EBV DNA and heavy smoker (LH), high EBV DNA and light smoker (HL), and high EBV DNA and heavy smoker (HH). Interestingly, there were significant differences among these four groups in terms of OS (93.1%, 87.2%, 82.9%, and 76.3%, *P*<0.001), PFS (87.0%, 84.0%, 73.9%, and 64.6%, *P*<0.001), DMFS (94.1%, 92.1%, 82.4%, and72.5%, *P*<0.001), and LRFS (92.8%, 92.4%, 88.7%, and 84.0%, *P*=0.012), respectively. Patients in the LL group (≤4000 EBV DNA copies and ≤6.5 pack/years) had better survival outcomes compared with either the LH group or HL group; however, the HH group experienced the poorest survival outcomes. Additionally, in the low EBV DNA groups, the cumulative effect of smoking did not remain an independent prognostic factor for OS (93.1% vs. 87.2%, *P*=0.176) or (87.0% vs. 84.0%, *P*=0.246). Conversely, in the high EBV DNA groups, the cumulative effect of smoking was confirmed as a significant prognostic factor for OS (82.9% vs. 76.3%, *P*=0.01) and PFS (73.9% vs. 64.6%, *P*=0.011; Figure 3).

In multivariate analysis, the combined classification was demonstrated to be an independent prognostic factor for OS (HR=1.39, 95%CI:1.20-1.60), PFS (HR=1.36, 95%CI:1.22-1.51), DMFS (HR=1.52, 95%CI:1.31-1.75), and LRFS (HR=1.32, 95%CI:1.13-1.55) after adjustment. In addition, the HRs for OS, PFS, DMFS and LRFS for the HH group were much higher than those observed when only baseline plasma EBV DNA was assessed. (Table 4)

**Table 4:** Combined prognostic value of the cumulative effect of smoking and baseline plasma EBV DNA for OS, PFS, DMFS and LRFS in the 1501 patients with NPC undergoing IMRT

Endpoint	Unadjusted HR	*P*-value*	Adjusted HR (95%CI)	*P*-value*
***OS***				
Low EBV	Reference		Reference	
High EBV	2.60(1.88-3.58)	<0.001	1.97(1.42-2.75)	<0.001
Low EBV, light smoker	Reference		Reference	
Low EBV, heavy smoker	1.45(0.84-2.48)	0.179	1.28(0.74-2.20)	0.374
High EBV, light smoker	2.43(1.61-3.67)	<0.001	1.88(1.23-2.87)	0.003
High EBV, heavy smoker	3.95(2.56-6.08)	<0.001	2.63(1.68-4.11)	<0.001
***PFS***				
Low EBV	Reference		Reference	
High EBV	2.26(1.78-2.87)	<0.001	1.91(1.49-2.45)	<0.001
Low EBV, light smoker	Reference		Reference	
Low EBV, heavy smoker	1.26(0.85-1.88)	0.248	1.18(0.79-1.75)	0.426
High EBV, light smoker	2.10(1.56-2.84)	<0.001	1.79(1.32-2.44)	<0.001
High EBV, heavy smoker	3.11(2.24-4.30)	<0.001	2.44(1.74-3.41)	<0.001
***DMFS***				
Low EBV	Reference		Reference	
High EBV	3.09(2.23-4.27)	<0.001	2.55(1.82-3.55)	<0.001
Low EBV, light smoker	Reference		Reference	
Low EBV, heavy smoker	1.39(0.79-2.44)	0.251	1.36(0.78-2.38)	0.284
High EBV, light smoker	3.09(2.05-4.66)	<0.001	2.58(1.70-3.91)	<0.001
High EBV, heavy smoker	4.11(2.64-6.41)	<0.001	3.28(2.09-5.16)	<0.001
***LRFS***				
Low EBV	Reference		Reference	
High EBV	1.68(1.18-2.39)	0.004	1.68(1.18-2.39)	0.004
Low EBV, light smoker	Reference		Reference	
Low EBV, heavy smoker	1.05(0.60-1.86)	0.86	1.26(0.69-2.29)	0.452
High EBV, light smoker	1.52(0.98-2.34)	0.61	1.52(0.99-2.36)	0.058
High EBV, heavy smoker	2.10(1.29-3.40)	0.003	2.51(1.49-4.23)	0.001

Abbreviations: CI = confidence interval; DMFS = distant metastasis-free survival; EBV= Epstein-Barr virus deoxyribonucleic acid; HR = hazard ratio; IMRT = intensity-modulated radiotherapy; LRFS = locoregional relapse-free survival; OS = overall survival.

Low EBV is a baseline plasma EBV DNA load ≤ 4000 copies/mL; high EBV, > 4000 copies/mL. Light smoker is a smoking index ≤ 6.5 pack-years; heavy smoker, > 6.5 pack-years.

* The following parameters were included in the Cox proportional hazards model multivariate analysis with backward elimination: age (> 45 vs. ≤ 45 years), gender (female vs. male), T category (T3–4 vs. T1-2), N category (N2–3 vs. N0-1), chemotherapy (yes vs. no) and the combination of cumulative effect of smoking and baseline plasma EBV DNA.

## DISCUSSION

Having witnessed the revolutionary changes that IMRT has provided in the treatment of NPC in recent decades, we consider it necessary to reevaluate the value and significance of existing prognostic factors. Cigarette smoking has already been reported to play an important role in EBV latent-to-lytic activation [13]; therefore, we sought to investigate the combined prognostic value of cigarette smoking and baseline plasma EBV DNA in patients with NPC undergoing IMRT. To the best of our knowledge, this is the first large population study to evaluate the combined prognostic value of these factors in an attempt to refine risk stratification and prognostication in NPC.

In this study, both baseline plasma EBV DNA and cigarette smoking were significant prognostic factors for survival outcomes. Interestingly, although baseline plasma EBV DNA had superior prognostic value, the cumulative effect of smoking correlated positively and complemented the prognostic value of baseline plasma EBV DNA, leading to improved risk stratification and prognostication. The combined prognostic value of both factors was more significant than the individual factors and may enhance the traditional TNM classification, suggesting that more intensive treatment should be recommended for high-risk patients with NPC identified by combined assessment of baseline plasma EBV DNA and cigarette smoking.

The prognostic significance of cigarette smoking has previously been investigated in NPC. Ouyang et al. [14] revealed that cigarette smoking was prognostic for poorer OS, PFS, DMFS and LRFS in NPC, with the risk increasing with the exposure to smoking, with cut-off points of 32 pack-years for OS and 22 pack-years for PFS. Chen et al. [12] reported that that smoking was prognostic for poorer outcomes in male patients, and patients with smoking index≤15.5 pack-years had better OS, PFS, DMFS and LRFS. Guo et al. [15] indicated smoking could increase the risk of locoregional recurrence in NPC. However, in our study, heavy smokers had poorer OS, PFS and DMFS, but not LRFS. This difference is probably due to the excellent local control rates (> 90%) achieved by the introduction of IMRT and the fact that distant metastasis, rather than local failure, is the predominant pattern of failure in the IMRT era. [16] On the other hand, the differences observed between this study and previous studies also reflect the necessity of reevaluating existing prognostic factors for NPC in patients undergoing IMRT.

In recent studies, baseline plasma EBV DNA was found to positively correlate with the tumor burden and was closely associated with TNM staging, survival, recurrence and prognosis. [17-19] Despite its wide clinical use, baseline plasma EBV DNA alone was far from adequate, considering the heterogeneity of patients with NPC. Here, we further explored the complementary role of cigarette smoking in combination with baseline plasma EBV DNA. Interestingly, cigarrete smoking correlated positively and complemented baseline EBV DNA to improve risk stratification and prognostication in NPC. We created a novel prognostic model, in which patients were divided into four risk groups. Significant differences in OS, PFS, LRFS and DMFS were observed between the four groups: patients in the LL group had better survival outcomes than both the LH group and HL group, and the HH group experienced the poorest survival outcomes. In particular, heavy smokers had a poorer prognosis than light smokers in the high EBV DNA group; however, a similar trend was not observed in the low EBV DNA group. These results were not unexpected. Xu et al. [13] reported that cigarette smoke extract promoted EBV replication *in vitro* and induced expression of immediate-early transcriptional activators, which subsequently increased the transcription of BFRF3 and gp350 in the lytic phase, which are responsible for the EBV latent-to-lytic switch. As cigarette smoking promotes EBV latent-to-lytic activation, these findings may help to explain our result that smoking status had more significant prognostic value in the high EBV DNA group than that in low EBV DNA group. In other words, cigarette smoking had more specific and peculiar prognostic value for poorer outcomes in patients with high EBV DNA, whereas the diverse outcomes of patients with low EBV DNA may be attributed to other unknown factors. Future studies are required to identify other potential prognostic factors for NPC. Other evidence also indicates that smoking exacerbates tissue hypoxia and leads to smoking-induced tissue hypoxia. [20] Also, inhalation of cigarette smoke reduced tumor control after RT in animal models, [21] which could lead to a poorer outcome in heavy smokers. However, the results of this study indicate cigarette smoking does not have significant prognostic value in patients with low baseline plasma EBV DNA. Future research is necessary to explore the associated mechanisms.

The major objective of this study was to reevaluate the prognostic value of both cigarette smoking and baseline plasma EBV DNA and investigate the combined prognostic value of these factors for risk stratification and prognostication in patients with NPC receiving IMRT. The improved prognostic ability offered by combining cigarette smoking and baseline plasma EBV DNA with the TNM staging system could enable more aggressive treatment protocols for patients with a poorer prognosis and conversely, allow patients with a better prognosis to undergo less intensive treatment strategies. According to the guidelines of the National Comprehensive Cancer Network (NCCN) version 1.2015, definitive RT of the nasopharynx and elective RT of the neck is recommend as the standard treatment regimen for stage I NPC, While for stage II and locoregionally-advanced NPC, CCRT with adjuvant chemotherapy was recommended as a category 2A evidence, and CCRT alone as a category 2B evidence. [22, 23] In addition, recently several multicentre randomised controlled trials have indicated that cisplatinum-based induction chemotherapy has been shown to improve disease-free survival and may be considered in locally advanced disease. [24] Based on this study, it is possible that current chemoradiotherapy may be not sufficient for patients with high baseline plasma EBV DNA who smoke heavily, even those with an early TNM classification. For patients in this risk stratification, additional NACT followed by CCRT may be an optimal choice. Moreover, patients with low baseline plasma EBV DNA who smoke lightly may not gain much benefit from neoadjuvant chemotherapy or adjuvant chemotherapy. Thus for this group of patients, CCRT protocols may be sufficient, especially for patients without lymphatic metastasis, which may avoid overtreatment and unnecessary side-effects. Further studies are required to identify a more reasonable systemic approach to improve the outcomes of patients in different risk stratifications.

The major limitation of this study is the fact it is a retrospective analysis of patients treated at a single cancer center in an NPC endemic area. Large scale, multi-institutional prospective studies are needed to confirm the findings of this research. Secondly, the smoking status of the patients at diagnosis was extracted from medical records, rather than using standardized questionnaires at enrollment.

In summary, both cigarette smoking and baseline plasma EBV DNA were independent prognostic factors for survival in patients with NPC undergoing IMRT. Combined assessment of baseline plasma EBV DNA and cigarette smoking refined risk stratification for specific patient subsets, especially patients with a high baseline plasma EBV DNA load. This prognostication method should be considered for inclusion in clinical decision-making regarding treatment strategies, and may further supplement the accuracy of the TNM classification system, refine risk stratification and guide the design of individualized treatment strategies to improve the treatment outcomes of patients with NPC receiving IMRT.

## MATERIALS AND METHODS

### Patient characteristics

All 1811 patients with newly-diagnosed, non-distant metastatic, histologically-proven NPC treated with IMRT at our institution between November 2009 and February 2012 were retrospectively reviewed. The patients recruited for the study completed a pretreatment evaluation, including physical examination, hematology and biochemistry profiles, quantitative analysis of baseline plasma EBV DNA by real-time quantitative polymerase chain reaction, magnetic resonance imaging (MRI) scan of nasopharynx and neck, chest radiography, abdominal sonography, and a single photon emission computed tomography (SPECT) whole-body bone scan; 29.2% (528/1811) of patients also underwent a (18)F-fluorodeoxyglucose (18F-FDG) positron emission tomography CT (PET/CT) examination. Before treatment, the following basic information was collected: age, sex, family history of NPC, smoking habits (including smoking status, packs of cigarette/day, years of smoking). Patients whose medical records were lacking data on either smoking habits or baseline plasma EBV DNA were excluded (*n* = 310). A total of 1501 patients were finally included in this study. All patients were restaged according to the 7^th^ edition of the UICC/AJCC staging system. This study was approved by the Institution of the Sun Yat-Sen University Cancer Center (SYSUCC). The clinicopathological characteristics of the patients are summarized in Table 1.

### Treatment strategies

The nasopharyngeal and neck tumor volumes of all patients were treated using radical RT based on IMRT for the entire course. Target volumes were delineated slice-by-slice on treatment planning CT scans using an individualized delineation protocol that complies with International Commission on Radiation Units and Measurements reports 50 and 62. The prescribed doses were 66–72 Gy at 2.12–2.43 Gy/fraction to the planning target volume (PTV) of the primary gross tumor volume (GTVnx), 64–70 Gy/28-33 fractions to the PTV of the GTV of the involved lymph nodes (GTVnd), 60-63 Gy/28-33 fractions to the PTV of the high-risk clinical target volume (CTV1), and 54–56 Gy/28-33 fractions to the PTV of the low-risk clinical target volume (CTV2). All targets were treated simultaneously using the simultaneous integrated boost technique. Institutional guidelines recommended only IMRT for stage I and concurrent chemoradiotherapy ± neoadjuvant/adjuvant chemotherapy for stage II to IVB. In this study, Overall,1306/1501 (87.0%) patients received chemotherapy. Of the total 1116 patients with locally-advanced NPC, 1057/1116 (94.7%) received chemotherapy. Reasons for deviation from the guidelines included individual patient's refusal, age, or organ dysfunction suggestive of intolerance to treatment. When possible, salvage treatments (intracavitary brachytherapy, surgery or chemotherapy) were provided for cases with documented relapse or persistent disease.

### Follow-up and study endpoints

Patients were followed-up from the first day of therapy to day of last examination or death. All patients were examined at least every 3 months during the first 2 years, and every 6 months for 3 years thereafter or until death. Median follow-up was 48.4 months (range, 1.3-76.4 months). The primary endpoint was overall survival (OS), defined as the time from start of treatment to death from any cause. The secondary endpoint was progression-free survival (PFS), defined as the time to tumor progression or death; distant metastasis-free survival (DMFS), defined as the time to tumor metastasis; and locoregional relapse-free survival (LRFS), defined as the time to the first locoregional relapse.

### Statistical analysis

We explored the association between survival outcomes and the combined prognostic value of baseline plasma EBV DNA and cigarette smoking. The cutoff value for baseline plasma EBV DNA was 4000 copies/mL. [7] Patients with baseline plasma EBV DNA > 4000 copies/mL were categorized into the high EBV DNA group and patients with baseline plasma EBV DNA≤4000 copies/mL, the low EBV DNA group. The cumulative effect of smoking was measured using the smoking index, which was calculated by multiplying cigarette packs per day and smoking time (years). Receiver operating characteristic (ROC) curve analysis was used to determine the optimal cut-off value; [25] the cut-off point for OS was 6.5 pack-years (0.42% sensitivity, 0.70% specificity, area under the curve of 0.55 [95% CI: 0.51-0.60; *P* < 0.028]). The associations between the two factors were examined using the Chi-square test or Fisher's exact test for nominal variables. For all endpoints, survival rates were calculated using the Kaplan-Meier method and survival curves were compared using the log-rank test. [26] Multivariate analyses were applied to determine the hazard ratios and assess independent significance using the adjusted Cox proportional hazards model with backward elimination. [27] Host factors (age, gender), tumor factors (T and N category), and treatment profiles (chemotherapy) were included as covariates. Statistical Product and Service Solutions (SPSS) version 19.0 software (IBM, Armonk, NY, USA) was used for all data analysis; two-tailed *P*-values < 0.05 were considered statistically significant.

## Abbreviations

AC: adjuvant chemotherapy
CCRT: concurrent chemoradiotherapy
CES: cumulative effect of smoking
CI: confidence interval
DMFS: distant metastasis-free survival
EBV DNA: Epstein-Barr virus deoxyribonucleic acid
HR: hazard ratio
IMRT: intensity-modulated radiation therapy
LRFS: locoregional relapse-free survival
MRI: magnetic resonance imaging
NACT: neoadjuvant chemotherapy
NPC: nasopharyngeal carcinoma
OS: overall survival
PET-CT: positron emission tomography–computed topography
PFS: progression-free survival
RT: radiotherapy
SI: smoking index
SPECT: single photon emission computed tomography
SYSUCC: Sun Yat-sen University Cancer Center
TNM: tumor-node-metastasis
UICC/AJCC: International Cancer Control/American Joint Committee On Cancer

## References

[1] Wei KR, Zheng RS, Zhang SW, Liang ZH, Ou ZX, Chen WQ (2014). Nasopharyngeal carcinoma incidence and mortality in China in 2010. Chinese journal of cancer.

[2] Zhang LF, Li YH, Xie SH, Ling W, Chen SH, Liu Q, Huang QH, Cao SM (2015). Incidence trend of nasopharyngeal carcinoma from 1987 to 2011 in Sihui County, Guangdong Province, South China: an age-period-cohort analysis. Chinese journal of cancer.

[3] Lee AW, Ma BB, Ng WT, Chan AT (2015). Management of Nasopharyngeal Carcinoma: Current Practice and Future Perspective. Journal of clinical oncology.

[4] Xu L, Pan J, Wu J, Pan C, Zhang Y, Lin S, Yang L, Chen C, Zhang C, Zheng W (2010). Factors associated with overall survival in 1706 patients with nasopharyngeal carcinoma: significance of intensive neoadjuvant chemotherapy and radiation break. Radiotherapy and oncology.

[5] Lin JC, Liang WM, Jan JS, Jiang RS, Lin AC (2004). Another way to estimate outcome of advanced nasopharyngeal carcinoma—is concurrent chemoradiotherapy adequate?. International journal of radiation oncology, biology, physics.

[6] Chan AT, Lo YM, Zee B, Chan LY, Ma BB, Leung SF, Mo F, Lai M, Ho S, Huang DP (2002). Plasma Epstein-Barr virus DNA residual disease after radiotherapy for undifferentiated nasopharyngeal carcinoma. Journal of the National Cancer Institute.

[7] Leung SF, Chan AT, Zee B, Ma B, Chan LY, Johnson PJ, Lo YM (2003). Pretherapy quantitative measurement of circulating Epstein-Barr virus DNA is predictive of posttherapy distant failure in patients with early-stage nasopharyngeal carcinoma of undifferentiated type. Cancer.

[8] Lin JC, Wang WY, Chen KY, Wei YH, Liang WM, Jan JS, Jiang RS (2004). Quantification of plasma Epstein-Barr virus DNA in patients with advanced nasopharyngeal carcinoma. The New England journal of medicine.

[9] Su WH, Chiu CC, Yao Shugart Y (2015). Heterogeneity revealed through meta-analysis might link geographical differences with nasopharyngeal carcinoma incidence in Han Chinese populations. BMC cancer.

[10] Hsu WL, Chen JY, Chien YC, Liu MY, You SL, Hsu MM, Yang CS, Chen CJ (2009). Independent effect of EBV and cigarette smoking on nasopharyngeal carcinoma: a 20-year follow-up study on 9,622 males without family history in Taiwan. Cancer epidemiology, biomarkers & prevention.

[11] Abdulamir AS, Hafidh RR, Abdulmuhaimen N, Abubakar F, Abbas KA (2008). The distinctive profile of risk factors of nasopharyngeal carcinoma in comparison with other head and neck cancer types. BMC public health.

[12] Chen C, Shen LJ, Li BF, Gao J, Xia YF (2014). Smoking is a poor prognostic factor for male nasopharyngeal carcinoma treated with radiotherapy. Radiotherapy and oncology.

[13] Xu FH, Xiong D, Xu YF, Cao SM, Xue WQ, Qin HD, Liu WS, Cao JY, Zhang Y, Feng QS (2012). An epidemiological molecular study of the relationship between smoking risk of nasopharyngeal carcinoma and Epstein-Barr virus activation. Journal of the National Cancer Institute.

[14] Ouyang PY, Su Z, Mao YP, Liang XX, Liu Q, Deng W, Xie FY (2013). Prognostic impact of cigarette smoking on the survival of patients with established nasopharyngeal carcinoma. Cancer epidemiology, biomarkers & prevention.

[15] Guo SS, Huang PY, Chen QY, Liu H, Tang LQ, Zhang L, Liu LT, Cao KJ, Guo L, Mo HY (2014). The impact of smoking on the clinical outcome of locoregionally advanced nasopharyngeal carcinoma after chemoradiotherapy. Radiation oncology.

[16] Jiang F, Jin T, Feng XL, Jin QF, Chen XZ (2015). Long-term outcomes and failure patterns of patients with nasopharyngeal carcinoma staged by magnetic resonance imaging in intensity-modulated radiotherapy era: The Zhejiang Cancer Hospital's experience. Journal of cancer research and therapeutics.

[17] Ma BB, King A, Lo YM, Yau YY, Zee B, Hui EP, Leung SF, Mo F, Kam MK, Ahuja A (2006). Relationship between pretreatment level of plasma Epstein-Barr virus DNA tumor burden metabolic activity in advanced nasopharyngeal carcinoma. International journal of radiation oncology, biology, physics.

[18] Lo YM, Leung SF, Chan LY, Lo KW, Zhang J, Chan AT, Lee JC, Hjelm NM, Johnson PJ, Huang DP (2000). Plasma cell-free Epstein-Barr virus DNA quantitation in patients with nasopharyngeal carcinoma. Correlation with clinical staging. Annals of the New York Academy of Sciences.

[19] Lo YM, Chan LY, Chan AT, Leung SF, Lo KW, Zhang J, Lee JC, Hjelm NM, Johnson PJ, Huang DP (1999). Quantitative and temporal correlation between circulating cell-free Epstein-Barr virus DNA and tumor recurrence in nasopharyngeal carcinoma. Cancer research.

[20] Jensen JA, Goodson WH, Hopf HW, Hunt TK (1991). Cigarette smoking decreases tissue oxygen. Archives of surgery.

[21] Grau C, Nordsmark M, Khalil AA, Horsman MR, Overgaard J (1994). Effect of carbon monoxide breathing on hypoxia and radiation response in the SCCVII tumor in vivo. International journal of radiation oncology, biology, physics.

[22] Chan AT (2011). Current treatment of nasopharyngeal carcinoma. European journal of cancer.

[23] Chen YP, Wang ZX, Chen L, Liu X, Tang LL, Mao YP, Li WF, Lin AH, Sun Y, Ma J (2015). A Bayesian network meta-analysis comparing concurrent chemoradiotherapy followed by adjuvant chemotherapy, concurrent chemoradiotherapy alone and radiotherapy alone in patients with locoregionally advanced nasopharyngeal carcinoma. Annals of oncology.

[24] Chan AT, Gregoire V, Lefebvre JL, Licitra L, Hui EP, Leung SF, Felip E, Group E-E-EGW (2012). Nasopharyngeal cancer: EHNS-ESMO-ESTRO Clinical Practice Guidelines for diagnosis, treatment and follow-up. Annals of oncology.

[25] Hanley JA (1989). Receiver operating characteristic (ROC) methodology: the state of the art. Critical reviews in diagnostic imaging.

[26] Hu XJ, Lagakos SW (2007). Nonparametric estimation of the mean function of a stochastic process with missing observations. Lifetime data analysis.

[27] Gill RD (1992). Multistate life-tables and regression models. Mathematical population studies.

